# Toward Primary Prevention of Cancer: The Case for a Global Strategy to Limit Avoidable Exposures

**DOI:** 10.1289/ehp.121-a137

**Published:** 2013-04-01

**Authors:** Valerie J. Brown

**Affiliations:** Valerie J. Brown, based in Oregon, has written for *EHP* since 1996. In 2009 she won a Society of Environmental Journalists’ Outstanding Explanatory Reporting award for her writing on epigenetics.

At least one-third of all cancer cases worldwide could be prevented, according to recent estimates. Tobacco use, poor diet, physical inactivity, and other behaviors are some of the preventable risk factors involved; other cancers have been attributed to environmental exposures such as outdoor air pollution and occupational hazards such as asbestos, metals, and substances used in consumer products. An international team of public and occupational health experts from the World Health Organization (WHO), universities, and independent agencies now makes a case for a global strategy for preventing these exposures [*EHP* 120(4):420–426; http://dx.doi.org/1205897].

The authors reviewed accounts of effective policies and interventions in PubMed as well as government and nongovernmental organization reports, and supplemented their data with recommendations from a WHO-sponsored conference held in March 2011. Most efforts now concentrate on secondary prevention and treatment, neglecting cost-effective primary prevention approaches, probably because the results are not easily quantifiable. Yet, the authors state, “Primary prevention that controls a common source of exposure to proven and probable carcinogens is far more effective and cost-effective than persuading thousands of persons to each change their individual behaviors.”

**Figure:**
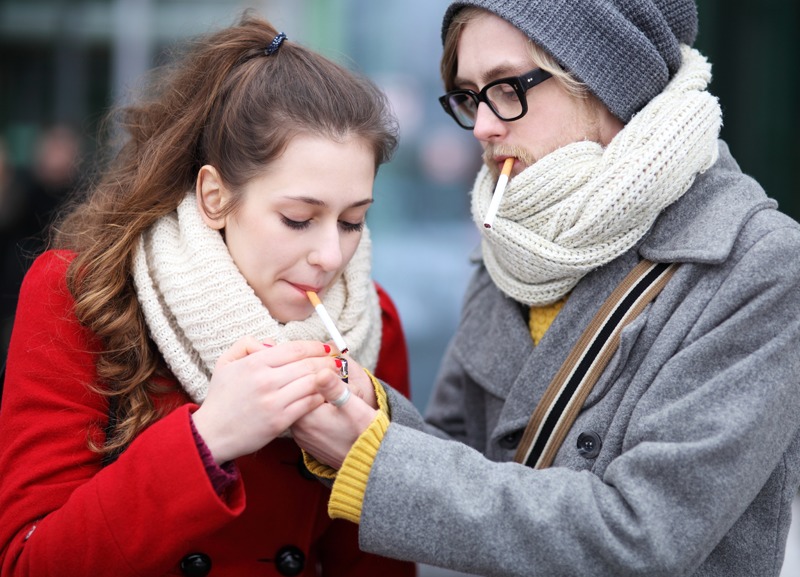
Persuading people to change their behaviors for health reasons is not always efficient or cost-effective. But primary prevention measures that address communal exposures, such as instituting smoke-free public areas, can protect a large number of people—while potentially encouraging individuals to make healthy lifestyle changes. © Edyta Pawlowska/Shutterstock

But avoiding exposures to many carcinogens can be difficult. The public and workers are often ignorant of both their exposures and ways to prevent them. Further difficulty arises from the long latency period for many cancers, which tends to obscure the connection between exposure to carcinogens and later health effects. The researchers emphasize that widespread “invisible” exposures such as persistent organic pollutants and substances in consumer products should be addressed through policies that work across sectors including housing, food, energy production, and industry. In other words, preventing more cancer cases depends on addressing more sources of a given carcinogen.

Yet, a broader perspective indicates there are many ways to reduce environmental risk factors, and some have multiple co-benefits, especially against noncommunicable diseases. For instance, the authors note that legislating smoke-free public places not only prevents secondhand cigarette smoke exposure but also encourages smokers to cut down or quit, potentially reducing cardiovascular disease risk. Likewise, reducing outdoor air pollution, particularly diesel exhaust, also reduces lung disease cases. What’s most needed, the authors say, is recognition that primary prevention is not only important but also feasible. With this recognition, as well as coordination of existing knowledge and tools, they believe primary prevention can become part of the policy framework for all governments.

